# Oncolytic measles virus therapy enhances tumor antigen-specific T-cell responses in patients with multiple myeloma

**DOI:** 10.1038/s41375-020-0828-7

**Published:** 2020-04-23

**Authors:** Nandakumar Packiriswamy, Deepak Upreti, Yumei Zhou, Rehan Khan, Amber Miller, Rosa M. Diaz, Cliona M. Rooney, Angela Dispenzieri, Kah-Whye Peng, Stephen J. Russell

**Affiliations:** 1 Department of Molecular Medicine, Mayo Clinic, Rochester, MN USA; 2 Division of Hematology, Mayo Clinic, Rochester, MN USA; 3 Vyriad Inc., Rochester, MN USA; 4 Center for Cell and Gene Therapy, Baylor College of Medicine, Houston, TX USA

**Keywords:** Phase I trials, Translational research

## Abstract

Oncolytic virus therapy leads to immunogenic death of virus-infected tumor cells and this has been shown in preclinical models to enhance the cytotoxic T-lymphocyte response against tumor-associated antigens (TAAs), leading to killing of uninfected tumor cells. To investigate whether oncolytic virotherapy can increase immune responses to tumor antigens in human subjects, we studied T-cell responses against a panel of known myeloma TAAs using PBMC samples obtained from ten myeloma patients before and after systemic administration of an oncolytic measles virus encoding sodium iodide symporter (MV-NIS). Despite their prior exposures to multiple immunosuppressive antimyeloma treatment regimens, T-cell responses to some of the TAAs were detectable even before measles virotherapy. Measurable baseline T-cell responses against MAGE-C1 and hTERT were present. Furthermore, MV-NIS treatment significantly (*P* < 0.05) increased T-cell responses against MAGE-C1 and MAGE-A3. Interestingly, one patient who achieved complete remission after MV-NIS therapy had strong baseline T-cell responses both to measles virus proteins and to eight of the ten tested TAAs. Our data demonstrate that oncolytic virotherapy can function as an antigen agnostic vaccine, increasing cytotoxic T-lymphocyte responses against TAAs in patients with multiple myeloma, providing a basis for continued exploration of this modality in combination with immune checkpoint blockade.

## Introduction

Multiple myeloma (MM) is a hematologic malignancy characterized by clonal expansion of bone marrow plasma cells. Up to 50% of patients achieve complete remission after initial treatment with high-dose melphalan and autologous hematopoietic stem cell transplant; yet, most of these patients relapse within 2–3 years because of the possible escape of MM cells [1, 2]. Numerous reports suggest that cytotoxic lymphocytes (CTLs) reactive against the escaping MM cells provide the best hope in efficiently clearing residual MM cells and paving the way to a durable complete remission [3]. MM cells express numerous tumor-associated antigens (TAAs), including cancer testis antigens, neoantigens, and differentiation antigens [3–6]. Cancer testis antigens expressed by malignant plasmablasts include MAGE-A1, MAGE-A2, MAGE-A3, NY-ESO-1, PRAME, MUC-1, WT-1, hTERT, p53, and SSX2 [4, 7]. While increased expression of TAA may promote myeloma cell survival and proliferation, it can also induce humoral and cell mediated antitumor immunity [6]. However, these host immune responses are usually not sufficient to control the tumor.

Various vaccination approaches are being evaluated in clinical trials to boost the host immune responses to known TAAs [5, 8, 9]. Of note, oncolytic viruses are being evaluated in this regard [10]. Oncolytic virotherapy provides a platform to target TAAs by mediating the inflammatory killing of infected myeloma cells, promoting the phagocytosis and cross presentation of released cellular proteins by tumor-resident antigen-presenting cells, and thereby enhancing antitumor cytotoxic T-cell responses. We have been investigating the potential of measles virus (MV), a lymphotropic negative sense RNA virus of the family *Paramyxoviridae*, as an oncolytic agent for MM [11]. MV-NIS is a recombinant MV (Edmonston strain) engineered to express the human thyroidal sodium iodide symporter (NIS) to facilitate noninvasive in vivo visualization of virus-infected cells using single-photon emission computed tomography. MV-NIS uses CD46 receptors to enter cells and to drive intercellular fusion of infected cells with their uninfected neighbors, resulting in formation of nonviable multinucleated syncytia [12]. Myeloma plasma cells overexpress CD46 and are therefore highly susceptible to MV-NIS killing [13]. We recently completed a Phase 1 dose escalation study evaluating the safety and maximal tolerated dose (MTD) of intravenous administration of a single dose of MV-NIS in patients with heavily pretreated relapsed refractory MM [14]. A total of 21 patients received the virus as a monotherapy (10^6^ to 10^11^ infectious units per patient) without encountering dose limiting toxicities, and the MTD was not reached (>10^11^ infectious units). ^123^I scans were positive in tumor deposits of four patients after virotherapy, confirming tumor-selective infection and replication of MV after intravenous delivery. One patient achieved a complete remission and less durable decreases in myeloma-specific IgG and/or serum free light chain (FLCs) levels were seen in other patients. Subsequently, additional patients were enrolled in an expansion cohort at the 10^11^ TCID_50_ dose level. In the present study, we investigated if MM patients receiving a single intravenous MV-NIS infusion had preexisting T-cell responses against ten different shared tumor antigens, and whether MV-NIS virotherapy enhanced their cytotoxic T-cell responses against these TAA.

## Materials and methods

### Patient samples

All patient samples used in the study originated from consenting MM patients enrolled in the phase I trial of systemic administration of MV-NIS (NCT00450814) [14]. In this study, we analyzed samples from patients who were treated with intravenous infusion of 10^11^TCID_50_ (50% tissue culture infectious dose) infectious units of MV-NIS. All study patients were scheduled for Peripheral blood mononuclear cell (PBMC) sample collections at predetermined on-study time points. However, the patients enrolled on this study had very advanced myeloma (median ten prior lines of therapy, typically including two autologous stem cell transplant procedures) resulting in low/variable PBMC yields and, in some cases, failure to comply with sample collection schedules or early transitioning to alternative therapy. Inclusion of patients in the current study was therefore dependent upon sample availability and patients with poor PBMC yields, or who did not comply with sample collections were excluded from the current analysis. PBMCs were isolated from heparinized blood collected before and at 6 weeks after virotherapy with use of density centrifugation over Ficoll within 4 h of collection. PBMCs were then cryopreserved in liquid nitrogen in medium containing 80% fetal bovine serum and 20% dimethylsulfoxide. Blood samples from healthy volunteers were purchased from Zen-Bio, Inc. (USA), processed and cryopreserved as PBMCs.

### Study oversight

This study was conducted in accordance with the principles of the Declaration of Helsinki. The protocol was approved by the local human investigations committee. Written informed consent was obtained from all the patients before screening.

### T-cell responses to tumor antigens

Interferon (IFN)-γ enzyme-linked immunospot (ELISPOT) assays were carried out in duplicate on PBMC samples to assess T-cell responses against selected tumor antigens for both time points (before and at 6 weeks after virotherapy). Previously frozen PBMCs were rested in vitro for a short period using an IL-7 plus IL-15 cytokine cocktail. Briefly, 5 × 10^6^ PBMCs were incubated in lymphocyte growth medium-3 (Lonza, USA) containing IL-7 (50 ng/mL) and IL-15 (25 ng/mL) at 37 °C for 48 h. Human interleukin (IL)-7 and IL-15 were purchased from PeproTech. Rested cells were then seeded at 2 × 10^5^ cells per well in capture antibody pre-coated 96-well ELISPOT plates. Cells were stimulated with TAA-specific peptide pools of overlapping 15-mer peptides spanning the entire amino acid sequence of each antigen (JPT Peptide Technologies Inc.). The test wells were incubated with MAGE-A1, MAGE-A3, MAGE-C1, WT-1, PRAME, SSX2, hTERT, p53, MUC-1, or NY-ESO-1 peptide mixes (2.5 μg of each peptide per mL). A CEF-positive peptide pool consisting of a mix of immunogenic peptides derived from cytomegalovirus, Epstein–Barr virus, and influenza viral antigens was included as a positive control. The ELISPOT plates were incubated at 37 °C for 48 h and were developed in accordance with manufacturer instructions (eBioscience, CA). Developed IFN-γ spots were counted with an automated ELISPOT reader (CTL Analyzers LLC, USA). Each spot represented a single reactive IFN-γ–secreting T cell. The frequencies of antigen-specific T cells were determined by subtracting mean spot counts for negative (DMSO) control wells from counts for test wells. T-cell responses in myeloma patients were considered positive when the mean adjusted counts were two-fold greater than the highest adjusted spot count observed in the healthy control cohort.

### RNA extraction and quantitative RT-PCR

CD138+ cells were isolated from five healthy volunteers and three unique MM patients PBMCs using EasySep™ Human CD138 Positive Selection Kit II (Stem cell Technologies, Vancouver, Canada). RNA was extracted using Qiagen RNeasy Mini kit (Qiagen, Germantown, MD). Reverse transcription was carried out with 1 μg of RNA with a cDNA synthesis kit (Promega, Madison, WI). Quantitative RT-PCR was performed as described previously for the expression ten TAA, and GAPDH [15]. Primers were obtained from IDT DNA Technologies. Primer sequences are listed in Supplementary Table. Quantitative RT-PCR was performed using LightCycler® 96 (Roche) and values were normalized to GAPDH.

### T-cell responses to measles viral antigens

T-cell responses against measles viral antigens hemagglutinin (H), nucleocapsid (N), fusion (F) were measured in a subset of patients. Briefly, overlapping peptides (15-mer with four offset) spanning the entire protein for MV H, N, and F proteins were synthesized (GeneMed Synthesis, Inc.). Patient PBMC samples were pulsed with measles-specific pepmixes in the presence on IL-7/IL-15 for 10 days and then re-stimulated for determination of measles-specific IFNγ production by ELISPOT. As a nonspecific viral antigen control, adenoviral hexon, and penton antigens were used.

### Neoantigen prediction and validation

Neoantigen prediction was performed as described before [16]. Briefly, DNA extracted from tumor cells and from normal PBMC was subjected to whole exome sequencing (WES) library preparation using SureSelect Target Enrichment System for Illumina Paired-End Sequencing Library Protocol (Agilent Technologies, Santa Clara, CA) with Clinical Research Exome capture oligo panel (Agilent Technologies). Barcoded WES libraries were pooled and sequenced on HiSeq 2500 (Illumina, San Diego, CA) in paired-end mode. HLA alleles were predicted using BWAkit algorithms with independent confirmation of allele typing using Polysolver (data not shown). Somatic mutations were called and sequences annotated by TGen. All somatic missense mutations in genes with protein products greater than or equal to 8 amino acids in length, excluding nonstop mutations, were used to produce potential neoantigen peptide sequences. Peptide tiling is performed by netMHC to identify all possible peptide sequences of 8, 9, and 10 amino acids containing the mutation by tiling a reading frame of corresponding length across the peptide sequence containing each somatic missense mutation. The corresponding wild-type genomic sequence was also used to generate wild-type consensus peptide sequences for later comparison. MHC class I binding affinity (half maximal inhibitory concentration, IC_50_) was predicted using netMHC for each HLA allele with every 8-, 9-, or 10-mer peptide generated from tiling mutant and corresponding wild-type peptide sequences. Binding affinity was predicted for each possible peptide: HLA allele combination. Following accepted standards of the field, IC_50_ < 500 nM was considered a predicted binder. Immune tolerance was considered possible when the wild-type consensus sequence had predicted binding affinity IC_50_ ≤ 500 nM to the same MHC class I molecule. Therefore, patient-specific neoantigens were defined as any unique combination of peptide sequence: HLA allele with mutant peptide binding affinity IC_50_ < 500 nM, and corresponding wild-type peptide IC_50_ > 500 nM. 8-,9-,10-mer peptides were synthesized (GeneMed Synthesis, San Antonio, TX) and functional validation of the predicted neoantigens was carried out using re-stimulation with patient-derived PBMCs. Neoantigens were pooled based on their physiochemical properties and pulsed with PBMCs in the presence of IL-7 (50 ng/mL) and IL-15 (25 ng/mL) containing LGM-3 media for 7 or 14 or 21 days. The cells were then collected and re-stimulated with neoantigen pools in the IFNγ coated ELISPOT plate. The plates were processed and developed as described earlier.

### Immunophenotyping of PBMCs

PBMCs (5 × 10^5^) were resuspended in 100 μL 1X phosphate buffered saline and stained with eFluor 455UV fixable live-dead stain (Thermo Fisher Scientific, Carlsbad, CA), to stain nonviable cells. The cells then were surface stained with anti-CD3, anti-CD4, anti-CD8, anti-CD45RA, anti-CCR7, anti-CD127, anti-CD25, and anti-PD1 fluorochrome conjugated antibodies (BioLegend, San Diego, CA) at room temperature for 30 min to identify the T-cell population and their memory or effector phenotypes. Cells were washed, and acquisition was carried out with a cell analyzer (BD LSRFortessaX-20; BD Biosciences, San Jose, CA). Data analyses were carried out with FlowJo data analysis software (FlowJo version 10.4.2, USA).

### Statistical analysis

Statistical analysis was performed using GraphPad Prism software (GraphPad Software v6, San Diego, CA). To determine differences between two independent (unpaired) groups, a nonparametric Mann–Whitney test was performed. Wilcoxon signed rank test of matched pairs was used to compare nonparametric paired data. The samples analyzed in our studies fell into three groups. Control healthy volunteer, patient pre-therapy samples and patient post-therapy samples. Control healthy volunteer samples and patient samples were independent and therefore unpaired. However, patient pre- and post-therapy samples are dependent and paired. To have a higher degree of stringency we considered the three groups as paired and also as unpaired and did both nonparametric Mann–Whitney *T* test and nonparametric Wilcoxon signed rank test. *P* value was calculated on both tests and was considered statistically significant only if *P* < 0.05 on both tests.

## Results

### Presence of T-cell responses against cancer testis proteins in patients with MM

**Fig. 1:**
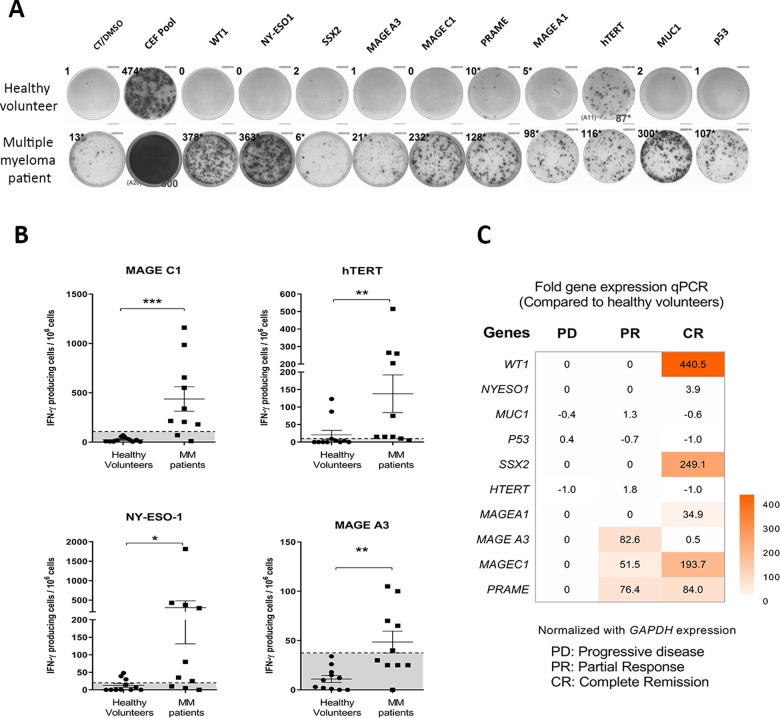
Patients with multiple myeloma react against tumor-associated antigens (baseline). **a** T-cell responses against ten tumor-associated antigens were compared between a healthy volunteer peripheral blood mononuclear cell (PBMCs) sample and PBMCs from a multiple myeloma patient with use of IFN-γ enzyme-linked immunospot (ELISPOT). The numbers of spots are listed above each well. CEF peptide pool was used as a positive control. TNTC indicates too numerous to count. **b** T-cell responses against MAGE-C1, hTERT, NY-ESO-1, and MAGE-A3 were significantly increased in patient PBMC samples (*n* = 10) compared with healthy volunteer PBMC samples (*n* = 10). Gray-shaded area denotes limit of negative response. **c** Heatmap showing fold change of TAA gene expression in CD138+ cells from MM patients (*n* = 3) compared with healthy volunteers (*n* = 5). Gene expression normalized to GAPDH.

To assess the basal T-cell responses of patient-derived PBMCs to ten selected TAAs, PBMC samples collected before virotherapy were compared with healthy volunteer PBMC samples using IFNγ ELISPOT assay. Even before virotherapy T-cell responses against TAAs were detected in most MM patients (Table 1 and Fig. 1a). There is no detectable TAA reactivity in T cells of healthy volunteers. In the ten myeloma patients analyzed, T-cell responses against MAGE-C1 and hTERT were detected in 80% of subjects; against NY-ESO-1 in 70%, MAGE-A3 in 50%, PRAME and SSX2 in 30%, p53 in 2, and WT-1 in only 10%. A patient with most positive T-cell responses prior to virotherapy is shown in Fig. 1a. Significantly stronger T-cell responses were present against NY-ESO-1, MAGE-A3, hTERT, and MAGE-C1 (*P* < 0.05) in this patient compared with samples from healthy volunteers (Fig. 1b). In addition, TAA gene expression in CD138^+^ cells from five different healthy volunteers and three unique MM patients (progressive disease, partial response, and complete response) as measured by quantitative RT-PCR showed expression of TAA in complete and partial response patients, but not in healthy volunteers (Fig. 1c).

**Table 1:** Positive T-cell responses against tumor-associated antigens before measles virotherapy.

Tumor-associated antigens	Number of patients had positive T-cell responses^a^, (*n* = 10)
MAGE-C1	8
hTERT	8
NY-ESO-1	7
MAGE-A1	6
MAGE-A3	5
MUC-1	5
PRAME	3
SSX2	3
p53	2
WT-1	1

^a^Compared with T-cell responses on peripheral blood mononuclear cell samples from healthy volunteers.

### MV-NIS therapy increased TAA-associated T-cell responses

**Fig. 2:**
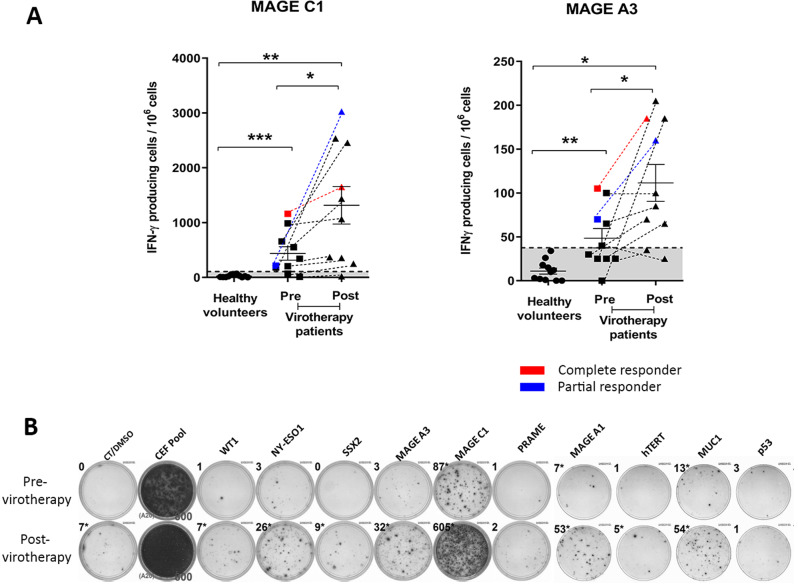
MV-NIS virotherapy induces increased T-cell reactivity against MAGE-C1 and MAGE-A3. **a** Of the ten tumor-associated antigens, T-cell responses against MAGE-C1 and MAGE-A3 were significantly increased in patient peripheral blood mononuclear cell samples after measles virus-sodium iodide symporter (MV-NIS) virotherapy. (*n* = 10). Gray-shaded area denotes the limit of negative response. **b** Representative image showing increased IFN-γ responses against MAGE-C1, MAGE-A1, and MUC-1 at 6 weeks after MV-NIS virotherapy. CEF peptide pool was used as a positive control. Sole asterisk indicates *P* < 0.05, double asterisks, *P* < 0.01; triple asterisks, *P* < 0.001, compared with the corresponding groups. Error bars indicate mean with standard error of mean. CEF indicates CEF-positive control peptide pools; CT/DMSO control/dimethylsulfoxide, IFN interferon.

We next tested whether oncolytic virotherapy induced or improved T-cell recall responses against the selected ten TAAs. We measured T-cell responses in patient samples collected before and 6 weeks after virotherapy. Of the TAAs tested, T-cell responses against MAGE-C1 and MAGE-A3 were significantly greater in post-virotherapy samples than pre-virotherapy samples (Fig. 2a). A patient with partial clinical response had the highest enhancement of T-cell response to MAGE-C1 shown in Fig. 2b.

### T-cell responses to TAA in a patient with sustained complete response to oncolytic virotherapy

**Fig. 3:**
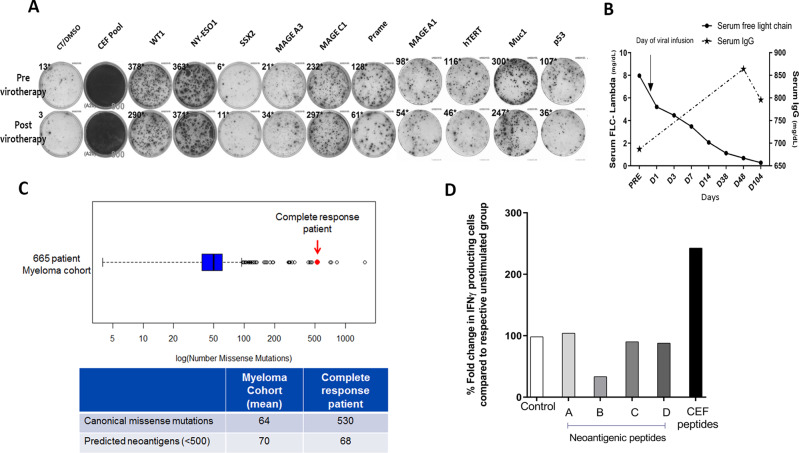
T-cell responses and mutational load in complete responder (CR) patient. **a** Complete remission patient, T-cell responses to most TAAs were detected pre and 6 weeks post virotherapy. **b** Serum FLC levels and Immunoglobulin (IgG) post-virotherapy in CR patient. FLC, free light chain; **c** Comparison of exome sequencing data on CR patient tumor and PBMCs samples revealed 530 somatic missense mutations. The patient in complete remission had a greater mutational load than a subset of 664 MM patients representing the CoMMpass population from the interim data of the Multiple Myeloma Research Foundation CoMMpass Study (NCT 01454297). **d** Functional validation of neoantigens using IFNγ ELISPOT revealed no T-cell response to the predicted 68 neoantigens. Neoantigens were pooled based on their physicochemical properties and tested for T-cell reactivity on 6 week post virotherapy sample. Each of the respective groups (A, B, C, D) was compared with their respected unstimulated groups and their % fold change is depicted. CEF peptide pool was used as positive control.

Among our ten patients who received 10^11^ TCID_50_ of MV-NIS, one patient had complete response, evidenced by the disappearance of tumors at multiple sites, complete resolution of monoclonal bone marrow plasmacytosis, and return of serum FLC (Fig. 3b) to reference levels [17] with corresponding increase in serum IgG levels. This complete remission was sustained for 9 months post virus infusion, at which point a focally relapsing frontal plasmacytoma was noted on PET/CT scan. This frontal lesion responded to local radiotherapy and complete disease remission was sustained for a further 15 months at which point a second focal relapse was noted, this time in a sternal plasmacytoma. Again, the focal sternal lesion was treated with local radiotherapy and, contrary to expectation; the patient has since remained in stringent complete remission without systemic relapse and without exposure to systemic antimyeloma therapy. Her PET/CT scans have remained negative and her most recent bone marrow aspirate 6 years post infusion of MV remains negative and shows no evidence of minimal residual disease by sensitive flow cytometry assay.

Our hypothesis to explain the sustained disease remission post virus administration in this patient, and the fact that neither of her focal recurrences were followed by systemic disease relapse, was that the virotherapy had boosted her antitumor T-cell response and that this response was further boosted after each of the local radiotherapy treatments due to the abscopal effect of radiotherapy, which has been well described in other malignancies [18–20].

Interestingly, analysis of T-cell responses in this patient showed high basal response to multiple TAA, which were sustained at all time points post virus administration, even years post virotherapy. However, the magnitude of these T-cell responses did not measurably change from baseline levels, neither after virotherapy (Fig. 3a).

To further investigate the possible role of cytotoxic T cells recognizing private tumor neoantigens (rather than shared TAA) in the maintenance of disease remission in this patient, we performed WES both on her tumor cells and normal lymphocytes to identify mutational burden. As a comparative analysis the mutational burden was compared with a cohort of 664 myeloma patients from Multiple Myeloma Research Foundation CoMMpass Database (NCT 01454297). The median mutational burden in 664 myeloma patients is 64 (reported earlier by our group) [16], and whereas the complete remission patient had a very high mutational burden with 530 mutations per genome (Fig. 3c).

We next analyzed the mutational data using an established neoantigen prediction algorithm and identified 68 potential neoantigens for the further study. Peptide pools corresponding to all of the identified neoantigens were synthesized and divided into four pools based on their differing predicted physicochemical properties. Stored PBMC samples from the patient were then tested by ELISPOT assay to determine cytotoxic T-cell frequencies against each of four peptide pools. However, no significant changes in the T-cell responses against any of the tested peptide pools were identified (Fig. 3d).

### T-cell responses to TAA associated with short-lived response or non-response to oncolytic virotherapy

**Fig. 4:**
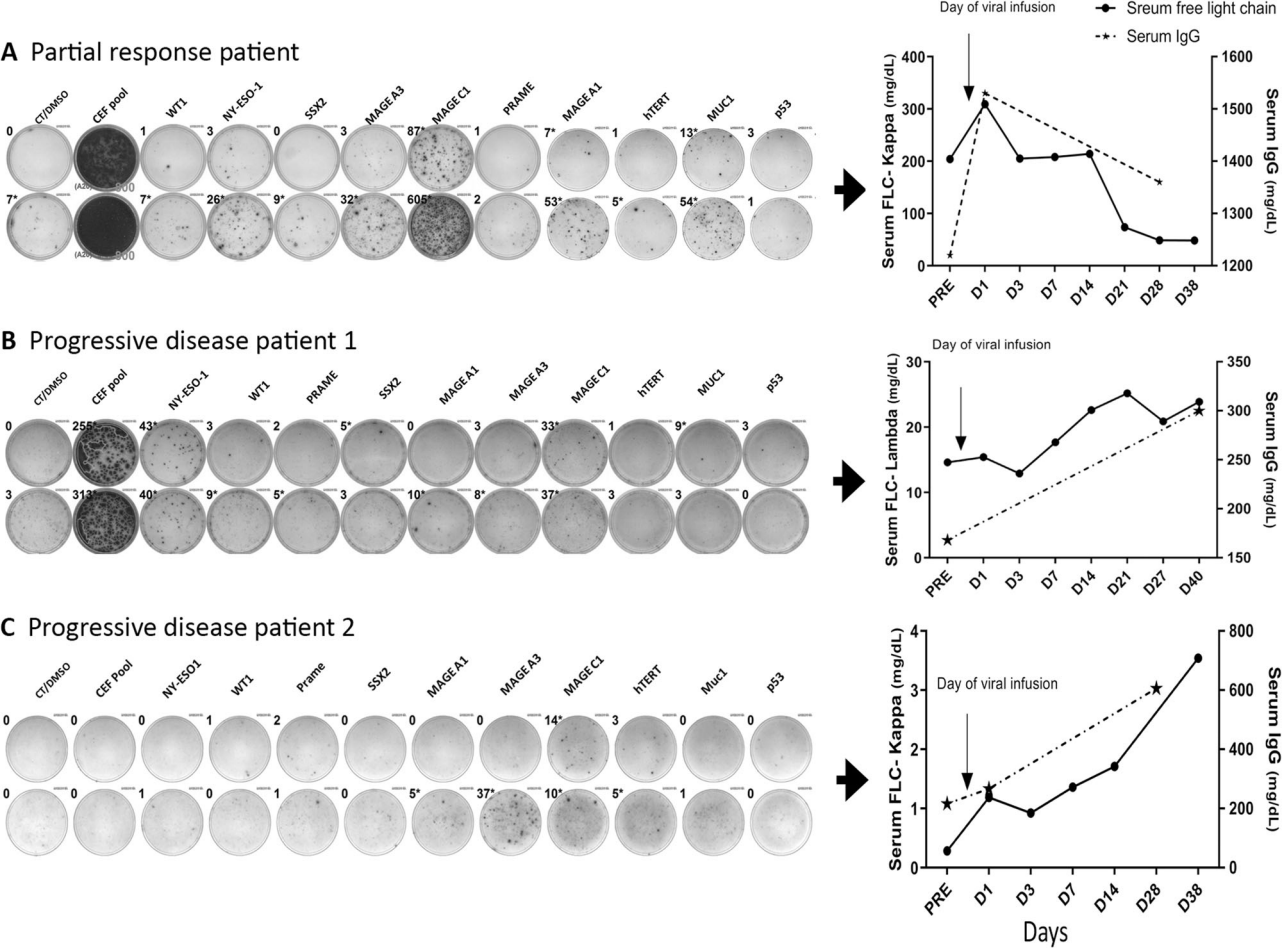
Identification of distinct T-cell response patterns to TAAs in MM patients after MV-NIS virotherapy. ELISPOT responses compared with serum FLC and IgG levels. **a** Partial response patient, T-cell responses to most TAAs were detected postvirotherapy only. This patient had a transient decline in serum FLC levels postvirotherapy. **b** Nonresponding patient, T-cell responses to TAAs were not detected before or after virotherapy. This patient had no decline in serum FLC levels post-therapy. **c** Nonresponding patient, T-cell responses to TAAs and CEF peptide pools were not detected before or after virotherapy. This patient had no decline in serum FLC levels post-therapy. CEF peptide pool was used as positive control. CT/DMSO control/dimethylsulfoxide, PRE previrotherapy, TAA tumor-associated antigen.

One patient had an incomplete response to virotherapy with immediate resolution of bone pain, greater than 50% reduction in the involved serum FLC, and a lesser decrease in the IgG monoclonal protein [17] (Fig. 4). This response was not sustained, lasting only 3 months following virus infusion, ELISPOT analyses on PBLs harvested from this patient pre- and post-virotherapy showed robust increases in cytotoxic T-cell responses to NY-ESO-1, MAGE-A3, MAGE-C1, MAGE-A1, and MUC-1 (Fig. 4a). Of the remaining eight patients, none responded clinically to the virus infusion, although some did show transient insubstantial reductions in the involved FLC. Post-virotherapy increases in cytotoxic T-cell responses were generally minor or absent in these patients (Fig. 4b, c).

### T-cell responses to measles antigens

**Fig. 5:**
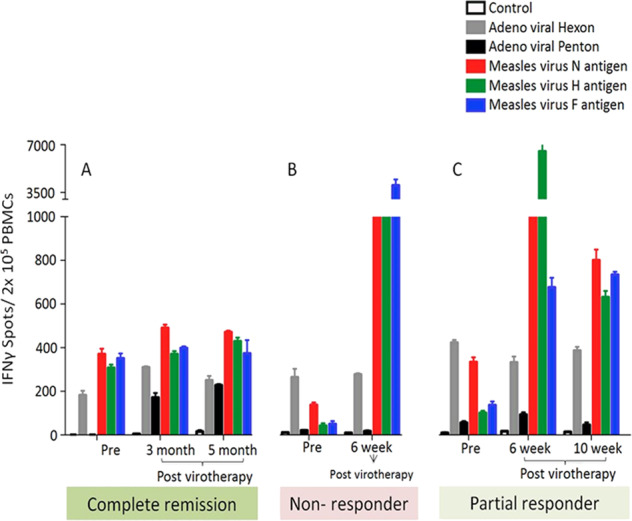
MV-NIS induced measles virus-specific T-cell responses in MM patients. 6 weeks post virotherapy T-cell responses against measles viral hemagglutinin (H), neuraminidase (N), and fusion (F) antigens were significantly higher in nonresponding (**b**) and partial response patient (**c**) but not in complete remission patient (**a**). Adenoviral penton and hexon were used as nonspecific viral antigen control.

T-cell responses to measles antigens pre- and postvirotherapy were measured in four of the myeloma patients who were seronegative at baseline. Data from three of the patients are shown in Fig. 5. Since these study subjects were measles seronegative at baseline, they were also expected to lack measles-reactive T cells. In line with this expectation, three of the patients analyzed had no detectable measles-reactive cytotoxic T cells at baseline, but developed antimeasles T-cell responses post-virotherapy, with parallel increases in their antimeasles antibody titers.

Interestingly, just one of the ten patients analyzed, the one who had a complete disease remission following measles virotherapy, showed robust antimeasles T-cell responses at baseline despite being measles seronegative. This patient also generated a very high titer of antimeasles antibodies post-therapy [13].

### Immune phenotyping of PBMC

**Fig. 6:**
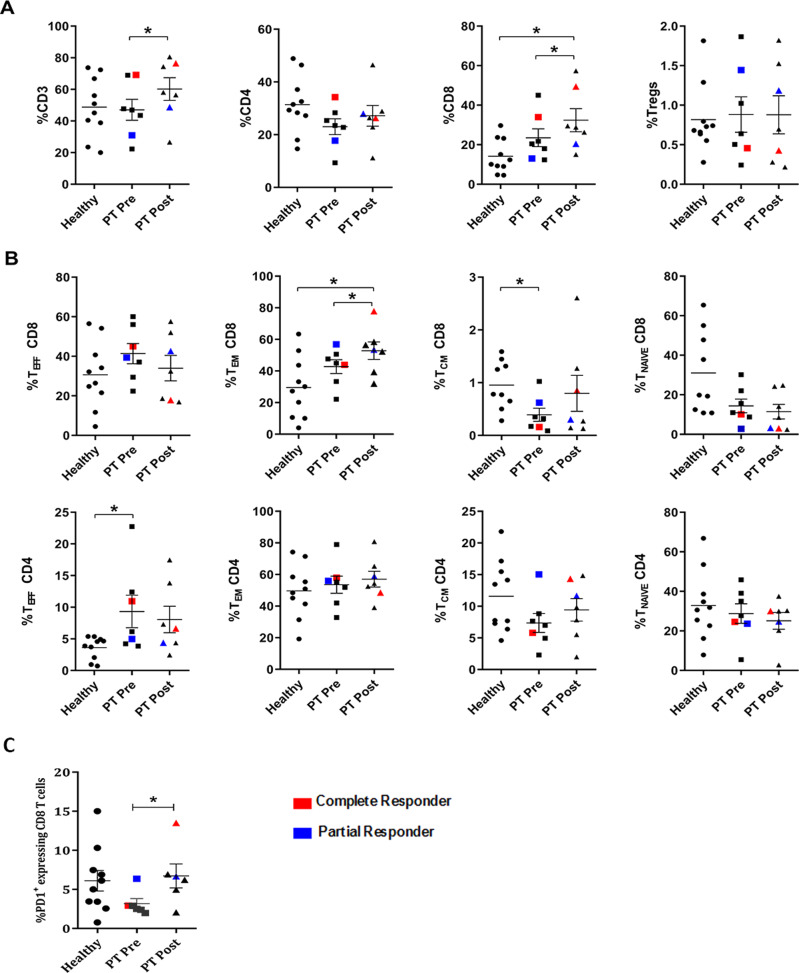
MV-NIS virotherapy induces changes in circulating CD8^+^ T cells. **a** Stored peripheral blood mononuclear cell samples from both patients and healthy volunteers were immune-phenotyped to determine T-cell percentages. Both %CD3^+^ and %CD8^+^ cells were found to be significantly increased in patients postvirotherapy. Complete responder is highlighted in red and partial responder in blue. **b** Percentages of the T-cell subpopulation based on expression of CCR7 and CD45RA. The number of CD4^+^ T effector cells was higher in patients before virotherapy. CD8^+^ T effector memory cells were more numerous among patients after virotherapy and healthy volunteers. CD8^+^ T central memory decreased in patients before virotherapy compared with healthy volunteers. **c** Percentages of PD1-expressing CD8^+^ T cells were greater in patients after virotherapy. Sole asterisk indicates *P* < 0.05; double asterisks, *P* < 0.01; triple asterisks, *P* < 0.001, compared with the corresponding groups. Error bars indicate mean with standard error of mean. Healthy indicates healthy controls; PT Pre patient pretreatment, PT Post patient posttreatment, T_CM_ central memory T cells, T_EFF_ effector T cells, T_EM_ effector memory T cells, T_Naïve_ naïve T cells, Tregs regulatory T cells.

To further investigate whether oncolytic virotherapy impacted the phenotype of circulating lymphocytes, we carried out immunophenotyping of PBMC. Samples from patients collected before and 6 weeks post virotherapy were analyzed by flow cytometry and compared with samples from normal donors. The pattern of CD45RA and CCR7 expression was used to define the proportion of naïve (CD45RA^+^CCR7^+^[T_Naïve_]), central memory (CD45RA^−^CCR7^+^[T_CM_]), effector memory (CD45RA^−^CCR7^−^[T_EM_]), and effector (CD45RA^+^CCR7^−^[T_EFF_]) cells within the CD4^+^ and CD8^+^ T-cell populations. Interestingly, MM patients treated with MV-NIS showed a significant increase in CD3^+^ T cells post-virotherapy compared with pre-virotherapy levels, and this was largely due to an increase in CD8^+^ T cells with no change observed in CD4^+^ T cells, nor in regulatory T cells (Fig. 6a).

In addition, sub-phenotyping of CD4 and CD8 T cells showed an increased proportion of CD8 T_EM_ among patients treated with MV-NIS (53%) compared with healthy donors (29%) and the previrotherapy levels (43%) (Fig. 6b). These data suggest a likely increase in antigen presentation and effector responses against tumor or viral antigens. The percentage of CD8 T_CM_ of MM patients before therapy was significantly lower compared with healthy donors, suggestive of depletion or immunosuppression. However, after MV-NIS therapy, the percentage of CD8 T_CM_ increased, suggesting that virotherapy had induced T-cell activation. Moreover, PD1 expression on CD8 T cells had increased significantly by 6 weeks post treatment compared with basal levels, indicating possible T-cell exhaustion, which is sequelae to the activation of CD8 T cells by virus-infected cells or released TAAs (Fig. 6c).

## Discussion

MM is a disseminated malignancy of neoplastic plasma cells that diffusely infiltrate the bone marrow and give rise to scattered bone marrow plasmacytomas (myelomas). In general the disease responds well to initial antimyeloma drug therapy, often remitting completely, but eventual relapse is almost inevitable, and the disease becomes progressively more treatment refractory and aggressive with each successive treatment-relapse cycle [21–23]. For these reasons, MM is not considered curable using currently available therapy.

Novel approaches to cancer immunotherapy have recently been shown to be capable of controlling and possibly curing certain types of cancer [6, 24] and are therefore being explored in MM. Myeloma cells do express a range of TAA and there have been numerous attempts to exploit this by generating and administering vaccines designed to provoke a therapeutic antimyeloma immune response. Popular targets have included myeloma patient-specific idiotypic immunoglobulin as well as a number of cancer testis antigens such as MAGE-C1, NY-ESO-1, MUC-1, and WT-1 [25, 26]. While these studies have provided proof of principle that antimyeloma CTL can be amplified using a variety of antigen-specific vaccine approaches, including the use of adjuvanted peptides and proteins, gene expression vectors, and antigen-pulsed dendritic cells, the clinical response rates have generally been underwhelming [27, 28]. Immune checkpoint inhibitor antibody therapy provides a potentially antigen agnostic approach to boost the T-cell responses not just to a variety of known myeloma TAAs, but also to unknown “neoantigens” which arise due to genomic instability and appear on the myeloma cell surface as class 1 MHC-mutated peptide complexes. However, randomized clinical trials have to date shown no benefit for immune checkpoint therapies in myeloma and have so often led to increased mortality (due to autoimmune disease) when combined with standard treatment of care [29–31].

An alternative approach to boost the immune response is through antigen agnostic oncolytic virotherapy. Oncolytic viruses represent a new class of therapeutic agents that promote antitumor responses through selective tumor cell killing and the induction of systemic antitumor immunity [10, 32–34]. Various genetically modified viruses have been developed as oncolytic agents, and in the present study we used a recombinant MV (Edmonston strain) encoding NIS to treat patients with MM. CD46 is the major cellular receptor for MV-NIS and is responsible for virus attachment entry and cell killing through cell–cell fusion [34, 35]. Transformed plasma cells from MM patients have been shown to express higher levels of CD46 than normal plasma cells [36]. Therefore, MV-NIS is an attractive new class of therapy for MM.

In our study, patients were treated not only with the aim of killing tumor cells but also to generate powerful and persisting antitumor immune stimulation. Induction of antitumor immune response occurs when professional antigen-presenting cells such as dendritic cells and macrophages ingest tumor antigens and migrate to the peripheral lymphoid tissues, present antigens along with a costimulatory signal, and activate naïve T lymphocytes. Thus, activated lymphocytes produce clones of antigen-specific effector cells that mediate the antitumor immune responses.

We measured T-cell responses of patients treated with oncolytic MV. Among the ten TAA-tested patients, we observed T-cell responses against MAGE-C1, hTERT, MAGE-A3, and NY-ESO-1 before virotherapy in more than 50% of patients. Yet, not all patients expressed the same TAA because of epidemiologic, biologic, and clinical heterogeneity, as described previously [37, 38]. In the present study, we also observed heterogeneity of TAA expression among different patients through direct quantitation of mRNA levels by RT-PCR (Fig. 1c) and indirectly by measuring T-cell responses before virotherapy (Fig. 1). We, for the first time, measured T-cell responses against common TAAs expressed in MM patients before and after oncolytic virotherapy. Interestingly, we observed an increase in T-cell responses against MAGE-C1 and MAGE-A3 after virotherapy, indicating that virotherapy may have antigen-agnostically enhanced the adaptive immune response to these antigens. We note that clinical response was associated with an increase in T-cell responses to TAA, whereas clinical non-response was associated with diminished or absent T-cell responses to TAA. This suggests that the increase in T-cell responses to TAA is likely beneficial and may be attributable to the oncolytic virotherapy.

The complete disease remission seen in one of the patients enrolled in this study was reported earlier [14, 17]. This patient had rapid tumor regression accompanied by documented tumor-selective replication of MV-NIS, evidenced by NIS imaging. Additional unique characteristics were (1) a low baseline titer of antimeasles antibodies; (2) high baseline counts of both measles-reactive and tumor antigen-reactive T cells, and (3) a high mutational burden compared with 664 MM patients whose exome data are publicly available in the Multiple Myeloma Research Foundation CoMMpass Database (NCT 01454297) (Fig. 3c). At the time of writing this paper, five and a half years post therapy, this patient remains disease free having had no additional systemic myeloma therapy. However, she did have two focal relapses 9 months and 30 months post measles therapy, both of which were treated with local radiotherapy. We therefore speculate that the long-term remission observed in this patient is a consequence of the sustained immune control of residual myeloma cells, likely driven by their high mutational burden, causing greater expression of tumor neoantigens that were targeted by cytotoxic T cells. We further speculate that the tumor-reactive cytotoxic T cells were additionally boosted by the abscopal effect of the radiotherapy she later received. While our attempts to document an increased post-measles frequency of neoantigen-specific CTLs were unsuccessful, we are inclined to attribute this to the inadequacy of current antigen prediction algorithms. Also, based on exome sequencing analysis, the haplotype of this patient was found to be HLA-A*03:01, HLA-B07:02, and HLA-C07:02 and we have not so far been able to identify appropriate haplotype-specific tetramer reagents for further exploration of this question. Interestingly and in contrast to most other malignancies, it was previously reported that increased mutational burden in myeloma negatively correlates with disease-free survival [16]. A possible explanation is that conventional myeloma therapies are generally immunosuppressive and therefore negate any potential positive impact of the immune system on disease outcome.

One of the most important conclusions of our study is that an oncolytic virus can antigen-agnostically boosts the T-cell response to TAA. In particular, T-cell responses against MAGE-C1 and MAGE-A3 were significantly amplified after MV-NIS virotherapy in a number of patients. In addition to the measles viral antigens, NIS encoded within MV could also be immunogenic. Studies have shown the presence of auto antibodies against NIS in a subset of autoimmunity induced thyroiditis patients [39] indicating NIS could act as antigen. In mouse tumor models expressing human NIS (hNIS), repeated DNA vaccination with hNIS induced NIS associated CD4 and CD8 T-cell immune response along with improved efficacy [40]. It is likely that at least in mouse tumor models hNIS (86.2% sequence similarity with mouse NIS) [41] could act as foreign antigen and induce hNIS-specific T cells. In our current study, we did not look at T-cell response against hNIS, however; it will be interesting to look at the magnitude of T-cell response against NIS after virotherapy and will be a subject of future studies.

Cross priming of CD8^+^ T cells is inefficient in the absence of antigen-specific CD4^+^ T cells, which in turn are activated and driven to proliferate when they encounter cognate class II MHC-peptide complexes on appropriately activated antigen-presenting cells [42, 43]. In the current study, we captured both CD4^+^ and CD8^+^ T-cell recall responses by using peptide mixes derived from a scan of 15-mer peptides with an overlap of 11 amino acids for each TAA but the assay was not designed to discriminate the different T-cell populations. PBMC immune-phenotyping data did show that several patients had a significant increase in CD4^+^ effector T cells counts before virotherapy, but there was no discernible post-therapy increase suggesting that MV-NIS may preferentially impact CD8 positive antitumor CTLs.

MM patients treated with MV-NIS had a significant increase in their percentage of circulating CD3^+^ and CD8^+^ T cells post-virotherapy. This result is in keeping with the previously reported increase in CD3^+^ and CD8^+^ T cells following intravenous oncolytic reovirus virus therapy in patients with a range of different advanced cancers [44]. Upon more detailed analysis of the circulating T cells we were able to document increases in effector memory CD8 (CD45RA^−^CCR7^−^CD8^+^), central memory CD8^+^ (CD45RA^−^CCR7^+^CD8^+^), and effector memory CD4^+^ (CD45RA^−^CCR7^−^CD4^+^) cells. These results suggest that specific memory CD8^+^ and CD4^+^ cells had been generated before virotherapy and responded anamnestically to the TAA released from virus-infected tumor cells. However, further studies will be necessary to confirm the specificity of these T-cell subsets. In addition, tetramer staining to identify TAA-specific T cells could have been used to validate our findings from ELISPOT. However, the lack of haplotype-specific tetramer reagents and limited availability of PBMC samples limited further testing.

Our findings are particularly encouraging for the broader application of oncolytic virotherapy because MM is an intrinsically immunosuppressive malignancy, which causes profound hypogammaglobulinemia, and reprograms the tumor microenvironment to subvert tumor-associated macrophages and dendritic cells to a protumoral, immunosuppressive, phenotype. Immune responses are further suppressed in MM patients by conventional antimyeloma drug therapy, especially with alkylators, corticosteroids, and proteasome inhibitors. In addition, responsiveness to immunotherapy has been linked to high mutational burden, hence high antigenic load, and MM cells generally have only a small number of mutations per genome [16, 45].

In contrast to “conventional” approaches to tumor vaccination, oncolytic virotherapy provides a potentially self-tuning stimulus to the antimyeloma immune response. Following oncolytic virotherapy, intratumoral propagation, and virus-mediated tumor cell killing continue until local inflammatory and systemic immune responses have amplified sufficiently to eliminate the virus infection. In parallel, the immune system is continuously exposed to tumor antigens that have been released by dying tumor cells, processed by antigen-presenting cells, and presented in conjunction with viral antigens.

While tumor responses were not achieved in the majority of patients enrolled in the current study, our findings do support the conclusion that oncolytic virotherapy can amplify the antimyeloma T-cell response and that under certain circumstances this can lead to long-term tumor control. We conclude that oncolytic virotherapy is a highly promising approach to myeloma immunotherapy and should be aggressively pursued in combination with other immunomodulatory approaches.

## Supplementary information

Supplementary Information
