# Supplementary material for: Oncolytic measles virus therapy enhances tumor antigen-specific T-cell responses in patients with multiple myeloma
Source: Leukemia. 2020 Apr 23;34(12):3310–22. doi: 10.1038/s41375-020-0828-7 (PMC7581629; doi:10.1038/s41375-020-0828-7)
Supplement: Supplementary file 1 — Supplementary Information [file 41375_2020_828_MOESM1_ESM.docx]

**Supplementary table.** Real time qPCR primer sequences for TAA and GAPDH genes.

| **Antigen** | **Primer pairs** | **5' to 3'** |
| --- | --- | --- |
| **NYESO1** | Forward | GTGTCCGGCAACATACTGACT |
|  | Reverse | GCCAAAAACACGGGCAGAAA |
| **PRAME** | Forward | CAAGCGTTGGAGGTCCTGAG |
|  | Reverse | ATCGGCTCTGAATGGAACCC |
| **MAGE A1** | Forward | AGTAGTAGGTTTCTGTTCTATTGGG |
|  | Reverse | TACTTATTCCACTGCTGTTATTATCC |
| **MAGE A3** | Forward | TCTTCAGCAAAGCTTCCAGTTC |
|  | Reverse | CAGCAGGCCATCGTAGGAGA |
| **MAGE C1** | Forward | GGCATTTTGTGACGAGGATCG |
|  | Reverse | CTCACAGGTCGCCTGTCTTC |
| **hTERT** | Forward | GGAGCAAGTTGCAAAGCATTG |
|  | Reverse | TCCCACGACGTAGTCCATGTT |
| **P53** | Forward | CAATAGGTGTGCGTCAGAAGC |
|  | Reverse | TACATCTCCCAAACATCCCTCAC |
| **WT1** | Forward | CAGGCTGCAATAAGAGATATTTTAAGCT |
|  | Reverse | GAAGTCACACTGGTATGGTTTCTCA |
| **SSX2** | Forward | TCATGTGTAATAAACGGGCCGA |
|  | Reverse | GAGCCTGCCGAAAGTCATCT |
| **MUC1** | Forward | ACCATCCTATGAGCGAGTACC |
|  | Reverse | GCCACCATTACCTGCAGAAAC |
| **GAPDH** | Forward | AGCTTGTCATCAATGGAAATCCC |
|  | Reverse | GTGAAGACGCCAGTGGACTC |
